# The association of Malnutrition-Inflammation Score with sleep quality and mental health in hemodialysis patients: a multicenter cross-sectional study

**DOI:** 10.1186/s12882-025-04089-0

**Published:** 2025-07-01

**Authors:** Mehrnaz Morvaridi, Hossein Bavi Behbahani, Meysam Alipour, Ahmad Zare Javid, Sara Keramatzadeh, Shiva Shokri, Pardis Tofighzadeh, Fatemeh Fayazfar, Haleh Soltaniyan Dehkordi, Elahe Ghadimi, Siavash Babajafari Esfandabad, Shokouh Shayanpour

**Affiliations:** 1 Department of Nutrition, School of Public Health, Iran University of Medical Sciences, Tehran, Iran; 2 Department of Nutrition, Shoushtar Faculty of Medical Sciences, Shoushtar, Iran; 3 Nutrition and Metabolic Diseases Research Center Clinical Sciences Research Institute, Ahvaz Jundishapur University of Medical Sciences, Ahvaz, Iran; 4 Student Research Committee, Ahvaz Jundishapur University of Medical Sciences, Ahvaz, Iran; 5 Student Research Committee, School of Nutrition and Food Sciences, Shiraz University of Medical Science, Shiraz, Iran; 6 Student Research Committee, Shoushtar Faculty of Medical Sciences, Shoushtar, Iran; 7 Nutrition Research Center, School of Nutrition and Food Science, Shiraz University of Medical Science, Shiraz, Iran; 8 Department of Internal Medicine, Chronic Renal Failure Research Center, Ahvaz Jundishapur University of Medical Sciences, Ahvaz, Iran

**Keywords:** Hemodialysis, Malnutrition, Inflammation, Sleep quality, Mental health

## Abstract

**Background:**

Hemodialysis is crucial for individuals with end-stage renal disease (ESRD), but it poses challenges that impact health and quality of life. Malnutrition, inflammation, sleep disturbances, and mental health disorders are common among hemodialysis patients, interconnecting and influencing outcomes. Therefore, the study aimed to investigate the association between the Malnutrition-Inflammation Score (MIS) and various health parameters, including sleep quality and mental health in hemodialysis patients.

**Methods:**

A multicenter cross-sectional study investigated the association between the MIS and health parameters in 423 hemodialysis patients across eight centers. Assessments included MIS, physical activity levels, dietary intake, sleep quality, and mental health status, along with biochemical parameters. Statistical analyses using SPSS software were conducted to identify associations.

**Results:**

Significant differences were observed in demographic, clinical, and biochemical characteristics across MIS quartiles (*p* < .05). Older age, lower BMI, longer dialysis vintage, and altered biochemical profiles were noted in higher MIS quartiles. Depression scores were significantly higher in higher MIS quartiles (*p* = .04). Multivariable analyses confirmed these associations, indicating higher odds of poorer sleep quality, depression, and anxiety with increasing MIS quartiles (*p* < .05).

**Conclusion:**

The study highlights the intricate association between malnutrition, inflammation, sleep quality, and mental health conditions in hemodialysis patients. Addressing these factors comprehensively and tailoring interventions may lead to improved outcomes and well-being.

## Introduction

Hemodialysis represents a critical life-sustaining therapy for individuals grappling with end-stage renal disease (ESRD), albeit accompanied by a plethora of challenges that profoundly affect patients’ health and overall quality of life [1]. Among these challenges, malnutrition, inflammation, sleep disturbances, and mental health disorders stand out as prevalent and interconnected factors that significantly impact the well-being of hemodialysis patients. Understanding the intricate interplay among these factors holds paramount importance in enhancing patient care and striving for improved outcomes within this vulnerable population [2–4].

Malnutrition and inflammation are frequent occurrences in hemodialysis patients and are closely linked to adverse clinical consequences, including heightened morbidity and mortality rates [5]. The Malnutrition-Inflammation Score (MIS) has emerged as an invaluable instrument for evaluating the nutritional and inflammatory status of these individuals, furnishing clinicians with crucial insights into their disease burden and prognostic indicators [6, 7]. Despite its extensive exploration concerning nutritional and inflammatory markers, the association of MIS with other pivotal health outcomes, such as sleep quality and mental health conditions, remains relatively uncharted territory [8–10].

Sleep disturbances represent a prevalent issue among hemodialysis patients, with studies reporting rates of insomnia, sleep fragmentation, and excessive daytime sleepiness ranging from 50 to 80% [11–13]. These disruptions stem from diverse factors, encompassing uremia, electrolyte imbalances, fluid overload, comorbidities such as obstructive sleep apnea, and treatment-related variables like dialysis schedule and medication effects [14–16]. Diminished sleep quality not only contributes to fatigue and impaired daytime functioning but also exacerbates pre-existing health conditions, thereby diminishing the overall quality of life for hemodialysis patients [17, 18].

Furthermore, mental health disorders, particularly anxiety and depression, are highly prevalent in this patient cohort, with reported rates significantly surpassing those observed in the general population. These conditions are intricately linked to heightened mortality risk, reduced treatment adherence, and diminished quality of life [19–21]. The etiology of mental health disorders in hemodialysis patients is multifaceted, encompassing biological, psychological, and social determinants. Chronic illness burden, perceived stress, social isolation, and the psychosocial ramifications of renal failure all contribute to the development and exacerbation of mental health conditions in this population [22–24].

Several studies have investigated sleep quality as well as mental health issues in hemodialysis patients [25–27]. One study identified a negative correlation between MIS and sleep quality among individuals with Restless Leg Syndrome (RLS) [28], while another associated depressive symptoms in hemodialysis patients with diminished quality of life and an increased symptom burden [29]. Furthermore, cancer patients undergoing hemodialysis exhibit higher rates of depression, poorer quality of life, and sleep disturbances, though the association with MIS is only a weak correlation in cancer patients [30].

Hemodialysis patients experience a complex interplay of malnutrition, inflammation, psychological disorders, and sleep disturbances, all of which significantly impact their health and quality of life. Emerging evidence suggests that these issues often coexist and may influence each other through shared biological and behavioral pathways [1, 31, 32]. For instance, chronic inflammation associated with malnutrition can disrupt neurotransmitter function and contribute to psychological disorders such as depression and anxiety [33]. Similarly, poor sleep quality, a common problem in dialysis patients, has been linked to systemic inflammation and worsened nutritional status [34, 35]. Moreover, psychological issues such as depression can exacerbate malnutrition by reducing appetite and motivation for self-care [36, 37]. Despite these interconnections, few studies have explored whether these conditions occur simultaneously, sequentially, or through bidirectional interactions, particularly in the context of the malnutrition-inflammation complex [32, 35, 38].

Understanding these relationships is critical to developing targeted, holistic interventions for this vulnerable population. Therefore, this study aims to investigate the associations between the MIS, sleep quality, and mental health conditions in hemodialysis patients, providing insights into their simultaneous occurrence and interdependence. By elucidating these associations, our aim is to enrich the burgeoning literature on the comprehensive care of hemodialysis patients and furnish evidence-based recommendations for clinical practice. Ultimately, our findings harbor the potential to ameliorate patient outcomes, enhance quality of life, and guide the development of personalized interventions aimed at optimizing the health and well-being of individuals undergoing hemodialysis.

## Methods

### Study design and setting

This research employed a multicenter cross-sectional study design to investigate the association between the MIS and various health parameters, including sleep quality and mental health conditions, among hemodialysis patients. The study encompassed eight hemodialysis centers located across three cities: Ahvaz, Shiraz, and Shushtar, Iran. These centers represented a diverse mix of healthcare settings, including both governmental and private institutions, ensuring a broad representation of the hemodialysis patient population. This study was conducted according to the guidelines laid down in the Declaration of Helsinki and all procedures involving human subjects were approved by the ethics committee of the Ministry of Health and Medical Education of Iran and the Shoushtar Faculty of Medical Sciences in Shoushtar, Iran (approval number: IR.SHOUSHTAR.REC. 1403.019). Written informed consent was obtained from all subjects.

### Participants

The study focused on adult patients aged 18 years and older who had undergone regular hemodialysis treatment for at least six months, ensuring an adequate duration of exposure to hemodialysis for the assessment of chronic health conditions and their correlations with the MIS. Hemodialysis was performed two or three times per week, with each session lasting approximately four hours, using high-flux dialyzers and bicarbonate-based dialysate. Patients received standard anemia management protocols, including intravenous iron supplementation and erythropoietin-stimulating agents (ESAs). Intravenous iron (iron sucrose) was administered based on individual iron status assessed through serum ferritin and transferrin saturation levels. ESAs were provided subcutaneously at dosages tailored to achieve target hemoglobin levels, adjusted according to the clinical response and ESA resistance index.

Inclusion criteria included age greater than 18 years and receiving regular hemodialysis for a minimum of six months. Exclusion criteria encompassed patients who had received enteral or parenteral feeding to avoid confounding nutritional status, those with cognitive or communication impairments to ensure accurate data collection, individuals who had been diagnosed with severe neurological or mental disorders to maintain study focus, those with active neoplastic disease to mitigate potential confounding effects on inflammation and nutritional status, patients with severe alcohol or drug addiction that could influence outcomes, individuals with major amputations to exclude those with significant physical limitations, those diagnosed with cancer, acute or chronic pancreatitis, or irritable bowel syndrome due to their potential impact on inflammation and nutritional status, and patients with hepatic insufficiency to maintain homogeneity within the study population.

### Data collection

Data were collected through patient interviews and review of medical records. To ensure the reliability of medical records, only records that had been updated within the past three months were used for data extraction. For key variables such as laboratory results and medication history, consistency checks were performed between the medical records and patient-reported data. Interviews were conducted by a trained dietitian using standardized protocols to minimize interviewer bias and ensure consistency across participants. Combining medical records and patient-reported data may introduce bias due to differences in data accuracy and completeness. To address this, cross-referencing was performed for overlapping variables (e.g., medication usage, weight changes) between the two sources. Discrepancies were resolved by consulting with the dialysis unit’s healthcare team.

Height was measured by having participants remove their shoes and any headwear. They were then positioned against a wall with their heels, buttocks, and shoulders touching the surface. Participants were instructed to look straight ahead with arms naturally by their sides. The measuring arm or slider was lowered until it firmly touched the top of their head in a neutral position, and the height was recorded to the nearest 0.1 centimeter (cm). Changes in weight over the past 6 months were collected from patient files. The patient’s weight was assessed in two states: once before dialysis and once after dialysis, with the weight after dialysis being used to calculate BMI. For weight measurement, participants were asked to remove heavy outer clothing and shoes. They were directed to stand on the scale platform, distributing their weight evenly on both feet. Participants were instructed to maintain a natural posture with arms at their sides. The weight displayed on the scale was recorded to the nearest 0.1 kg (kg). BMI was calculated using the formula: BMI = weight (kg) / (height (m))^2^.

In this study, a comprehensive assessment protocol was implemented to evaluate various aspects of participants’ health, including their nutritional and inflammatory status. The Malnutrition-Inflammation Score (MIS) is a validated tool designed to assess the dual burden of malnutrition and inflammation in hemodialysis patients. It is widely recognized for predicting clinical outcomes such as hospitalization, morbidity, and mortality. The MIS consists of 10 components across four domains: medical history (dietary intake changes, gastrointestinal symptoms, functional capacity), physical examination (muscle wasting, subcutaneous fat loss), body mass index (BMI), and laboratory parameters (serum albumin, total iron-binding capacity, and other markers). Each component is scored from 0 (normal) to 3 (severely abnormal), with a total MIS ranging from 0 to 30. Higher scores indicate more severe malnutrition and inflammation. Participants were categorized into three groups: normal nutritional status (MIS 0–7), mild to moderate malnutrition (MIS 8–18), and severe malnutrition (MIS 19–30) [39, 40].

Dietary intake was assessed through interviews conducted by a trained dietitian using the validated 168-item Food Frequency Questionnaire (FFQ) [41]. The dietitian followed a standardized protocol to ensure accuracy and consistency during data collection:

- Training: The dietitian received training on using the FFQ and standardized portion size estimation techniques.
- Interview Procedure: Each participant underwent a one-on-one interview. The dietitian guided participants in recalling their typical food consumption over the previous six months, focusing on food items included in the FFQ.
- Portion Size Estimation: Participants were shown standard measuring tools (e.g., cups, spoons, models, and photos of food portions) to accurately estimate portion sizes.
- Frequency Recording: Participants were asked to report the frequency of consumption for each food item, categorized into daily, weekly, or monthly intake.
- Conversion to Nutrient Data: The dietitian converted portion sizes into grams using standard food composition tables (Iranian Food Composition Table and USDA Food Composition Database) and calculated nutrient intakes using Nutritionist-4 software for dietary analysis.
- Validation: Responses were cross-checked with participants to ensure accuracy, particularly for items contributing significantly to protein and calorie intake.

The FFQ collected data on the frequency and portion size of various food items consumed over the previous six months. The reported frequencies were standardized to daily intake values. Portion sizes were converted to grams using standard food composition tables, including the Iranian Food Composition Table (IFCT) and the USDA Food Composition Database for missing items. Macronutrient and calorie intakes were calculated in grams/day using these converted values. The total energy and macronutrient intakes were recorded and analyzed. Protein intake was calculated as grams per kilogram of body weight (g/kg BW), and calorie intake was expressed as kilocalories per kilogram of body weight (kcal/kg BW) based on the participants’ post-dialysis weights.

Sleep quality and disturbances were evaluated using the Pittsburgh Sleep Quality Index (PSQI), which examined multiple domains including sleep latency, duration, efficiency, and daytime dysfunction. The global PSQI score over 5 is considered as poor quality of sleep. Mental health status was assessed using the Depression Anxiety and Stress Scale 21 (DASS-21), a validated questionnaire measuring symptoms of depression, anxiety, and stress. the participants achieve scores of ≥ 10, ≥8, and ≥ 15, they were considered to have depression, anxiety, and stress respectively. Additionally, relevant biochemical parameters, such as serum albumin levels, were extracted from the dialysis unit’s database to complement clinical assessments and provide further insights into participants’ health status and disease severity. The International Physical Activity Questionnaire (IPAQ) was utilized to gauge participants’ physical activity levels and daily exercise habits.

### Data analysis

Data analysis was conducted using the Statistical Package for Social Sciences (IBM SPSS 24 Statistics, Armonk, USA). The normality of continuous variables was assessed using the Kolmogorov-Smirnov test and histogram visualizations. Continuous variables were presented as means ± standard deviations (SD), while categorical variables were presented as frequencies and percentages. Participants were categorized into quartiles based on their MIS scores. While the MIS score has predefined grading categories (normal: 0–7, mild-to-moderate malnutrition: 8–18, severe malnutrition: 19–30), we also categorized participants into quartiles based on their MIS scores to provide a more granular analysis. The quartile method allowed for a detailed exploration of trends across the full spectrum of MIS values, which is particularly useful in identifying subtle associations between MIS and health outcomes such as sleep quality and mental health. This approach complements the predefined grading categories by offering additional insights into the relationship between malnutrition-inflammation severity and the measured variables. Comparisons across MIS quartiles were performed using one-way ANOVA for continuous variables and chi-square tests for categorical variables.

Biochemical parameters were presented as mean ± SD. The MIS Score, Sleep duration, PSQI score, depression Score, anxiety score, and stress score were presented as mean ± SD, and the categories of MIS, Sleep quality, depression, anxiety and stress were presented as percentages for categorical variables. Multivariable logistic regression analyses were conducted to assess the association between MIS quartiles and health outcomes such as sleep quality, depression, and anxiety. To ensure the reliability of the findings and control for confounding variables, covariates were adjusted in a stepwise manner across three models: Model 0: Unadjusted (crude analysis), Model 1: Adjusted for demographic and clinical factors, including age, sex, physical activity, diabetes, hypertension, smoking status, job, marital status, education level, income status, inter-dialysis weight gain, dialysis vintage, dialysis time, frequency of hemodialysis sessions, fluid intake, and urine volume, and Model 2: Further adjusted for energy intake and prescribed medications such as erythropoietin and iron supplements. Odds ratios (ORs) and 95% confidence intervals (CIs) were calculated for each model, and p-values for linear trends were derived to evaluate the association across MIS quartiles. P-values for linear trends were used in logistic regression to compare the OR between different quarters (Q1to Q4). The lower quartile of the MIS score (Q1) was used as the reference category. Statistical significance was set at *p* < .05.

## Results

**Fig. 1:**
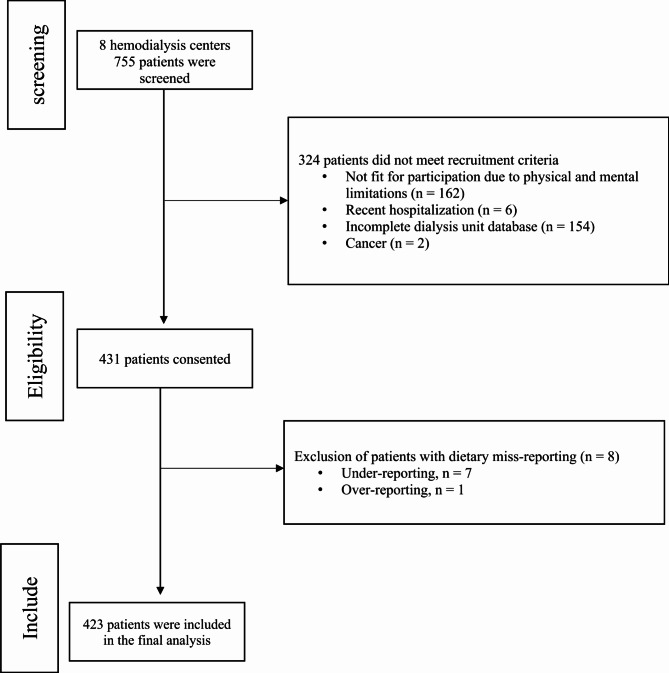
Study flowchart

Out of 755 patients screened across 8 hemodialysis centers, 268 patients were excluded from the study for various reasons, as shown in Fig. 1. Consequently, a total of 487 patients consented to participate in the study. Eight patients were further excluded from the final analysis due to dietary misreporting. Therefore, the final analysis included 423 patients. The characteristics of the participants in the study are outlined in Table 1. Key findings reveal significant differences across quartiles of MIS for various factors. Age exhibited significant variance (*p* = .002), with participants in Q4 displaying a higher mean age compared to those in Q1. BMI also demonstrated notable differences (*p* = .002), with participants in Q4 showing the lowest BMI compared to Q1. Dialysis vintage significantly differed (*p* < .001), with participants in Q4 having the longest duration compared to Q1. Similarly, dialysis time varied significantly (*p* < .001), with participants in Q4 having the longest duration compared to Q1. There was a significant discrepancy in urine volume less than 500 ml (*p* = .001), with higher proportions observed in Q4 compared to Q1. Education level also showed significant differences (*p* = .031), with a higher percentage of participants with less than 12 years of education in Q4 compared to Q1. Calcium carbonate and calcitriol prescription rates exhibited significant differences (*p* = .061 and *p* = .004, respectively), with higher mean doses observed in Q3 for calcium carbonate and in Q3 for calcitriol. Moreover, furosemide prescription frequency demonstrated significant variation (*p* = .021), with the highest mean dose in Q1 compared to Q4.

**Table 1:** Characteristics of participants in the study and associated factors across Malnutrition-Inflammation Score Quartiles

Characteristics, mean (SD) or *N* ()	MIS quartiles				*P* value*
	Q1(*N* = 108)	Q2(*N* = 100)	Q3(*N* = 104)	Q4(*N* = 111)	
Age, y	49.02 ± 14.09	53.04 ± 13.41	52.63 ± 14.94	56.57 ± 15.10	0.002
Male (n, %)	65 (60.2)	61 (61.6)	62 (60.2)	60 (54.1)	0.677
BMI, (kg/m^2^)	26.80 ± 4.38	25.88 ± 4.40	23.84 ± 5.01	22.92 ± 5.59	< 0.001
intradialytic weight gain, kg	1.82 ± 1.16	2.03 ± 1.05	2.05 ± 1.41	2.12 ± 1.05	0.263
PA, met-min/wk	373.78 ± 542.14	411.82 ± 1088.83	353.45 ± 1225.43	845.00 ± 4122.74	0.327
Dialysis vintage, month	17.78 ± 12.94	46.78 ± 51.35	58.60 ± 81.05	73.96 ± 64.33	< 0.001
Dialysis time, hours	3.52 ± 0.45	3.69 ± 0.55	3.70 ± 0.44	3.84 ± 0.50	< 0.001
Frequency dialysis per week, Time/week	2.85 ± 0.43	2.85 ± 0.43	2.94 ± 0.63	2.87 ± 0.56	0.544
Diabetes (n, %)	50 (46.3)	46 (46.0)	40 (38.5)	44 (39.6)	0.531
Hypertension (n, %)	84 (77.8)	72 (72.0)	81 (77.9)	79 (71.2)	0.529
Fluid intake, ml	1223.47 ± 1317.29	1291.28 ± 1030.79	1241.44 ± 775.63	1133.13 ± 881.85	0.723
**Urine volume**					
< 500 ml (n, %)	63 (58.3)	73 (73.0)	82 (78.8)	89 (80.2)	0.001
≥ 500 ml (n, %)	45 (41.7)	27 (27.0)	22 (21.2)	22 (19.8)	
**Center type**					< 0.001
Governmental (n, %)	36 (33.3)	61 (61.0)	80 (76.9)	94 (84.7)	
Private (n, %)	72 (66.7)	39 (39.0)	24 (23.1)	17 (15.3)	
**City**					< 0.001
Ahvaz (n, %)	78 (72.2)	45 (45.0)	35 (33.7)	26 (23.4)	
Shushtar (n, %)	20 (18.5)	30 (30.0)	11 (10.6)	8 (7.2)	
Shiraz (n, %)	10 (9.3)	25 (25.0)	58 (55.8)	77 (69.4)	
**Job**					0.244
Unemployed (n, %)	16 (14.8)	23 (23.0)	17 (16.3)	12 (10.8)	
Housekeeper (n, %)	33 (30.6)	30 (30.0)	37 (35.6)	45 (40.5)	
Retired (n, %)	24 (22.2)	24 (24.0)	27 (26.0)	23 (20.7)	
Employee (n, %)	13 (12.0)	5 (5.0)	4 (3.8)	6 (5.4)	
Self-employment (n, %)	14 (13.0)	13 (13.0)	15 (14.4)	21 (18.9)	
Others (n, %)	8 (7.4)	5 (5.0)	4 (3.8)	4 (3.6)	
**Marital status**					0.846
Married (n, %)	81 (75.0)	78 (78.0)	74 (71.2)	79 (71.2)	
Single (n, %)	22 (20.4)	16 (16.0)	22 (21.2)	22 (19.8)	
Divorced (n, %)	4 (3.7)	3 (3.0)	3 (2.9)	5 (4.5)	
Dead spouse (n, %)	1 (0.9)	3 (3.0)	5 (4.8)	5 (4.5)	
**Education**					0.031
< 12 years (n, %)	77 (71.3)	80 (80.0)	80 (76.9)	97 (87.4)	
≥ 12 years (n, %)	31 (28.7)	20 (20.0)	24 (23.1)	14 (12.6)	
**Income status**					0.640
< 5 million Rials (n, %)	31 (28.7)	41 (41.0)	31 (29.8)	31 (27.9)	
5–10 million Rials (n, %)	42 (38.9)	35 (35.0)	42 (40.4)	49 (44.1)	
10–20 million Rials (n, %)	29 (26.9)	19 (19.0)	23 (22.1)	24 (21.6)	
> 20 million Rials (n, %)	6 (5.6)	5 (5.0)	8 (7.7)	7 (6.3)	
**Medication prescriptions**					
Calcium carbonate 500 mg, time/day	1.03 ± 1.52	1.11 ± 1.64	1.55 ± 1.98	1.54 ± 2.01	0.061
Sevelamer hydrochloride 800 mg, time/day	1.09 ± 1.86	0.83 ± 1.44	0.86 ± 1.24	0.67 ± 1.28	0.212
Calcitriol 0.25 mcg, time/day	0.39 ± 0.87	0.62 ± 1.09	0.97 ± 1.36	0.73 ± 1.21	0.004
Furosemide, time/day	0.64 ± 1.28	0.31 ± 0.72	0.33 ± 0.79	0.32 ± 0.79	0.021
Corticosteroids (n, %)	6 (5.6)	1 (1.0)	6 (5.8)	3 (2.7)	0.208
Lipid-lowering drugs (n, %)	19 (17.6)	11 (11.0)	22 (21.2)	16 (14.4)	0.230
**Dietary intake**					
Energy, kcal/d	2357.92 ± 852.79	2226.84 ± 939.21	2248.51 ± 835.59	2119.81 ± 777.02	0.231
Energy, kcal/kg	32.01 ± 12.32	31.56 ± 13.17	34.49 ± 13.84	34.49 ± 14.36	0.233
Carbohydrates intake, g/d	355.70 ± 144.54	327.94 ± 145.14	338.13 ± 128.83	319.29 ± 126.34	0.234
Protein intake, g/d	87.05 ± 36.88	80.95 ± 37.97	77.52 ± 31.76	75.02 ± 30.42	0.059
Protein intake, g/kg	1.18 ± 0.54	1.14 ± 0.51	1.19 ± 0.51	1.22 ± 0.55	0.781
Total fat intake, g/d	67.59 ± 33.23	68.12 ± 39.20	68.15 ± 37.52	63.45 ± 35.09	0.735

Note: Data are means ± SD for quantitative variables and frequency (percent) for qualitative variables. Abbreviations: PA, physical activity; BMI, body mass index; MIS, Malnutrition-Inflammation Score. *Form ANOVA for quantitative variables and Chi-square for qualitative variables

The results presented in Table 2 highlight significant differences in various biochemical parameters across quartiles of MIS. Iron levels exhibited a significant difference (*p* = .001), with lower mean levels observed in Q4 compared to Q1. Ferritin levels also showed a significant difference (*p* = .01), with the highest mean levels in Q4 compared to Q1. Serum total iron binding capacity displayed a significant difference (*p* < .001), with decreasing mean levels observed from Q1 to Q4. Platelet counts exhibited a significant difference (*p* = .019), with lower mean counts observed in Q3 compared to Q1. Sodium levels showed a significant difference (*p* = .009), with lower mean levels in Q4 compared to Q1. Phosphate levels demonstrated a significant difference (*p* = .009), with decreasing mean levels observed from Q1 to Q4. Ca x P product levels displayed a significant difference (*p* = .012), with decreasing mean levels observed from Q1 to Q4.

**Table 2:** Biochemical parameters of participants in the study and associated factors across Malnutrition-Inflammation Score Quartiles

Characteristics, mean (SD) or *N* ()	MIS quartiles				*P* value*
	Q1(*N* = 108)	Q2(*N* = 100)	Q3(*N* = 104)	Q4(*N* = 111)	
FBG, mg/dL	114.16 ± 47.42	119.19 ± 59.22	109.95 ± 47.29	112.06 ± 48.84	0.603
HB, g/L	11.23 ± 1.84	10.97 ± 1.92	11.23 ± 1.91	11.43 ± 1.97	0.389
Iron, mg/dL	90.47 ± 83.98	70.99 ± 50.84	64.56 ± 42.51	62.76 ± 38.76	0.001
Ferritin, ng/mL	221.85 ± 230.89	259.48 ± 268.86	272.42 ± 256.7	332.8 ± 270.25	0.01
Serum total iron binding capacity, µg/dL	335.98 ± 73.66	329.76 ± 80.61	276.98 ± 93.01	259.31 ± 105.11	< 0.001
Hematocrit,	34.88 ± 7.15	34.38 ± 5.31	34.47 ± 6.11	35.05 ± 7.07	0.853
Platelets, 10*3/µg	205.05 ± 63.31	192.75 ± 66.67	177.21 ± 60.75	184.96 ± 75.67	0.019
Creatinine, mg/dL	8.74 ± 4.46	8.16 ± 3.18	7.91 ± 4.38	7.41 ± 2.74	0.070
Calcium, mg/dL	8.45 ± 0.95	8.39 ± 0.92	8.38 ± 0.89	8.36 ± 0.83	0.880
Sodium, mmol/L	140.43 ± 6.16	138.31 ± 4.49	139.95 ± 4.43	138.99 ± 4.26	0.06
Potassium, mmol/L	4.94 ± 0.77	5.06 ± 0.74	5.04 ± 0.91	5.15 ± 0.87	0.338
Phosphate, mg/dL	5.46 ± 1.11	5.30 ± 1.58	4.99 ± 1.26	4.92 ± 1.42	0.009
Ca x P, mg^2^/dL^2^	46.03 ± 9.74	44.49 ± 14.09	42.06 ± 11.43	41.13 ± 12.9	0.012
Albumin, g/ dL	4.23 ± 0.38	4.19 ± 0.78	5.96 ± 19.51	3.94 ± 0.54	0.407

Note: The data are presented as “mean ± SD”. Abbreviations: FBG, fasting blood glucose; HB, hemoglobin; Ca, calcium; P, Phosphorus. *The significant difference based on One-way ANOVA (*P* < .05)

The association between higher MIS scores and older age, lower BMI, and altered biochemical profiles (e.g., lower TIBC) reflect the components of the MIS assessment tool itself, rather than the identification of novel associations. These findings confirm the utility of the MIS as a composite marker of malnutrition and inflammation rather than a standalone diagnostic tool for independent association.

The results in Table 3 provide insights into mental health conditions and their association with MIS quartiles among the study participants. The MIS score displayed a significant difference (*p* < .001) across quartiles, with progressively higher mean scores observed from Q1 to Q4. The average MIS scores were 1.21, 3.41, 5.88, and 10.01 for quartiles Q1 to Q4, respectively (*p* < .001). Regarding nutritional status, 73.8% of patients were well-nourished, all of whom were in Q1, Q2, and Q3, with none in Q4 (*p* < .001). Mild-to-moderate malnutrition was observed in 25.1% of patients, exclusively in Q4. Severe malnutrition was present in 1.2% of the cohort, also only in Q4. Sleep duration did not exhibit a significant difference across quartiles (*p* = .92). The Pittsburgh Sleep Quality Index (PSQI) score, reflecting sleep quality, did not differ significantly across quartiles (*p* = .61). Depression score, as assessed by the Depression Anxiety Stress Scales (DASS) score, showed a significant difference (*p* = .04) across quartiles, with the highest mean score observed in Q3. Anxiety score, also assessed by DASS score, exhibit a significant difference across quartiles (*p* = .04). Stress score, assessed by DASS score, did not show a significant difference across quartiles (*p* = .20).

**Table 3:** Mental health conditions of participants in the study and associated factors across Malnutrition-Inflammation score quartiles

Characteristics, mean (SD) or *N* ()	TOTAL (*N* = 423)	MIS quartiles				*P* value*
		Q1(*N* = 108)	Q2(*N* = 100)	Q3(*N* = 104)	Q4(*N* = 111)	
**MIS**						
**MIS Score**	5.19 ± 3.55	1.21 ± 0.76	3.41 ± 0.49	5.88 ± 0.8	10.01 ± 2.14	< 0.001
**MIS category**						
Well-nourished (n, %)	312 (73.8)	108 (100)	100 (100)	104 (100)	0 (0)	< 0.001
Mild-to-moderate malnutrition (n, %)	106 (25.1)	0 (0)	0 (0)	0 (0)	106 (95.5)	
Severe malnutrition (n, %)	5 (1.2)	0 (0)	0 (0)	0 (0)	5 (1.2)	
**Sleep**						
**Sleep duration**	6.15 ± 2.52	6.15 ± 1.87	6.13 ± 1.9	6.28 ± 2.99	6.05 ± 3.03	0.92
**Sleep quality**						
**PSQI Score**	6.79 ± 3.67	6.3 ± 3.48	6.98 ± 3.85	6.88 ± 3.61	7.02 ± 3.75	0.44
**Sleep quality category**						
Good (n, %)	167 (39.5)	48 (44.4)	36 (36.0)	39 (37.5)	44 (39.6)	0.61
Poor (n, %)	256 (60.5)	60 (55.6)	64 (64.0)	65 (62.5)	67 (60.4)	
**Depression**						
**Depression Score**	13.39 ± 12.72	10.89 ± 11.94	15.48 ± 13.75	12.77 ± 12.03	14.5 ± 12.84	**0.04**
**Depression category**						
No (n, %)	196 (46.3)	58 (53.7)	42 (42.0)	51 (40.5)	45 (40.5)	0.17
Yes (n, %)	53.7 (22.7)	50 (46.3)	58 (58.0)	66 (59.5)	66 (59.5)	
**Anxiety**						
**Anxiety Score**	11.79 ± 11.49	9.59 ± 10.51	13.36 ± 14.43	12.13 ± 10.46	12.2 ± 10.1	0.10
**Anxiety category**						
No (n, %)	199 (47.0)	61 (56.5)	37 (37.0)	49 (47.1)	52 (46.5)	**0.04**
Yes (n, %)	224 (53.0)	47 (43.5)	63 (63.0)	55 (52.9)	59 (53.2)	
**Stress**						
**Stress Score**	16.58 ± 12.58	15 ± 12.42	18.68 ± 13.45	16.38 ± 11.98	16.41 ± 12.37	0.20
**Stress category**						
No (n, %)	223 (52.7)	61 (56.5)	48 (48.0)	56 (53.8)	58 (52.3)	0.66
Yes (n, %)	200 (47.3)	47 (43.5)	52 (52.0)	48 (46.2)	53 (47.7)	

Note: Data are means ± SD for quantitative variables and frequency (percent) for qualitative variables. Sleep status: good (PSQI score ≤ 4.99) or poor (PSQI score ≥ 5). Depression status: Yes (Depression score ≤ 9.99) or No (Depression score ≥ 10), Anxiety status: Yes (Depression score ≤ 7.99) or No (Depression score ≥ 8), Stress status: Yes (Depression score ≤ 14.99) or No (Depression score ≥ 15)

Note: Data are means ± SD for quantitative variables and frequency (percent) for qualitative variables. Abbreviations: MIS, Malnutrition-Inflammation Score, PSQI, Petersburg Sleep Quality Questionnaire

*Form ANOVA for quantitative variables and Chi-square for qualitative variables

Table 4 presents a multivariable logistic regression analysis for various variables across MIS quartiles. After adjusting for covariates, there was a significant trend (*p* = .02) indicating higher odds of poorer sleep quality with increasing MIS quartiles, with Model 2 showing the strongest association. Regarding depression, the odds ratios increased across MIS quartiles in Model 2, indicating a significant trend (*p* = .03) towards higher odds of depression with increasing MIS scores. Similarly, anxiety displayed a significant trend (*p* = .04) across MIS quartiles in Model 2, suggesting higher odds of anxiety with increasing MIS scores. However, stress did not show a significant trend across MIS quartiles in Model 2, although there was a trend towards higher odds of stress with increasing MIS scores (*p* = .10).

**Table 4:** Multivariable logistic regression analysis of the association between various variables and Malnutrition-Inflammation score (MIS) quartiles among the study participants

Variable	Q1 (*N* = 108)	Q2 (*N* = 100)	Q3 (*N* = 104)	Q4 (*N* = 111)	*P* for trend
**Sleep quality**					
Crude	Ref.	1.42(0.81,2.48)	1.33(0.77,2.31)	1.22(0.71,2.08)	0.53
Model 1	Ref.	1.82(0.97,3.42)	2.22(1.12,4.38)	2.30(1.11,4.78)	**0.03**
Model 2	Ref.	1.96(1.02,3.73)	2.32(1.15,4.68)	2.47(1.17,5.23)	**0.02**
**Depression**					
Crude	Ref.	1.6(0.93,2.77)	1.21(0.70,2.07)	1.7(1.00,2.91)	0.12
Model 1	Ref.	1.67(0.90,3.10)	1.33(0.68,2.60)	2.41(1.17,4.95)	**0.03**
Model 2	Ref.	1.61(0.86,3.03)	1.3(0.66,2.59)	2.42(1.16,5.06)	**0.03**
**Anxiety**					
Crude	Ref.	2.21(1.27,3.85)	1.46(0.85,2.5)	1.47(0.86,2.51)	0.36
Model 1	Ref.	2.16(1.13,4.14)	1.67(0.82,3.37)	2.24(1.06,4.72)	0.07
Model 2	Ref.	2.17(1.12,4.2)	1.75(0.86,3.58)	2.38(1.12,5.08)	**0.04**
**Stress**					
Crude	Ref.	1.41(0.81,2.43)	1.11(0.65,1.91)	1.19(0.7,2.02)	0.73
Model 1	Ref.	1.27(0.67,2.38)	1.43(0.71,2.87)	1.89(0.9,3.95)	0.09
Model 2	Ref.	1.16(0.6,2.23)	1.39(0.68,2.84)	1.87(0.87,4.03)	0.10

*P* < .05 statistically significant by multivariable logistic regression

Model 0. binary logistic regression analysis without adjustment

Model 1. binary logistic regression analysis with age, sex, physical activity, diabetes, hypertension, smoking, job, marital status, education, income status, inter-dialysis weight gain, dialysis vintage, dialysis time, frequency of hemodialysis sessions, fluid intake, and urine volume

Model 2. linear regression analysis with adjustment for model 1 in addition to energy intake, and medication prescriptions

## Discussion

Hemodialysis is a life-saving therapy for individuals with end-stage renal disease (ESRD), yet it poses numerous challenges that profoundly affect patients’ health and quality of life. In this study, we investigated the association between the Malnutrition-Inflammation Score (MIS) and various health parameters, including sleep quality and mental health conditions, in hemodialysis patients. Our results revealed significant associations between MIS quartiles and several key health outcomes, shedding light on the complex interplay among malnutrition, inflammation, sleep disturbances, and mental health disorders in this population.

### Association between MIS and demographic/clinical characteristics

Our analysis showed significant differences in demographic and clinical characteristics across MIS quartiles. Notably, participants in higher MIS quartiles tended to be older, with lower body mass index (BMI), longer dialysis vintage, and longer dialysis time. These findings are consistent with previous research demonstrating an increased risk of malnutrition and inflammation in older hemodialysis patients with longer disease duration [42, 43]. Additionally, the positive association between MIS score and dialysis vintage in our study was similar to the findings of previous studies [44, 45]. Furthermore, we observed differences in urine volume, education level, and prescription rates of certain medications across MIS quartiles, highlighting the heterogeneity of the study population and the multifaceted nature of their clinical presentations. It is important to note that these associations should not be interpreted as causal relationships but rather as observations reflecting the complexity of malnutrition and inflammation in hemodialysis patients.

The association between MIS and clinical characteristics such as age, BMI, and dialysis vintage are inherent to the construction of the MIS tool, which includes these parameters in its scoring system. Thus, the observed associations should be interpreted as validations of the MIS as a nutritional assessment instrument rather than the detection of new correlations. Since age and dialysis vintage are integral components of the MIS, their association with higher scores underscores the importance of considering these factors in patient management.

These demographic and clinical factors are integral components of the MIS scoring system and inherently influence its outcomes. For example, older patients and those with longer dialysis vintage are more likely to exhibit markers of chronic inflammation and nutritional decline, which could amplify their MIS scores [46]. This underscores the importance of considering these factors when interpreting MIS as an indicator of overall health status. The impact of these associations on the study’s conclusions is significant. First, the strong link between age and MIS highlights the need for age-specific nutritional and inflammatory interventions in hemodialysis patients [38]. Second, the association between longer dialysis vintage and higher MIS scores suggests that cumulative exposure to dialysis-related stressors contributes to worsening nutritional and inflammatory profiles [47]. These insights emphasize the importance of integrating longitudinal care strategies that address the evolving needs of patients over time. Finally, the influence of BMI on MIS scores highlights the potential limitations of using BMI alone as a nutritional marker, as it does not account for body composition changes, such as muscle wasting, which are common in this population [48, 49].

### Biochemical parameters across MIS quartiles

Analysis of biochemical parameters revealed significant differences across MIS quartiles, further underscoring the impact of malnutrition and inflammation on metabolic and physiological processes in hemodialysis patients. Participants in higher MIS quartiles exhibited lower levels of iron, ferritin, total iron-binding capacity, and platelet counts, indicating impaired iron metabolism and increased inflammatory activity. Ikeda-Taniguchi et al. established correlations between Functional Iron Deficiency (FID) and muscle wasting. They found that Total Iron-Binding Capacity (TIBC) serves as an independent indicator of muscle loss in hemodialysis (HD) patients, considering factors such as iron levels, inflammation, oxidative stress, and malnutrition [50]. Another study demonstrated that a decrease in baseline serum TIBC is associated with iron deficiency, Protein-Energy Wasting (PEW), inflammation, diminished Quality of Life (QoL), and mortality. Additionally, a decline in TIBC over time is independently linked to an increased risk of mortality [51]. Although our findings demonstrate strong associations between MIS and biochemical parameters, they do not imply a direct causal effect. Instead, they highlight the interrelationship between malnutrition, inflammation, and metabolic disturbances in hemodialysis patients.

The mechanism underlying these associations is multifaceted. Chronic inflammation, prevalent in hemodialysis patients with high MIS scores, disrupts iron homeostasis by increasing hepcidin levels, which impair iron absorption and mobilization. This results in Functional Iron Deficiency, where sufficient total body iron stores coexist with insufficient bioavailable iron for erythropoiesis, thereby exacerbating anemia [52]. Malnutrition further compounds these issues by reducing dietary iron intake and impairing the synthesis of proteins essential for maintaining muscle mass and overall health. Additionally, inflammation activates pro-inflammatory cytokines, such as IL-6 and TNF-α, which contribute to muscle breakdown and cachexia, leading to worsened clinical outcomes and increased mortality. These interrelated processes illustrate the significant metabolic burden faced by patients in higher MIS quartiles [1, 52, 53].

Participants in higher MIS quartiles had lower levels of sodium and phosphate, as well as higher levels of calcium-phosphate product, reflecting disturbances in mineral and electrolyte homeostasis commonly observed in hemodialysis patients. These associations suggest that poor nutritional status may contribute to altered mineral metabolism, but other confounding factors, such as dietary intake and dialysis efficiency, should be considered. Our findings align with the study by Mehrotra et al., which observed that malnourished dialysis patients (low serum albumin < 3.0 g/dL) had a lower incidence and severity of hyperphosphatemia compared to better-nourished patients. Their data suggest that reduced dietary protein intake, often associated with malnutrition, leads to lower serum phosphate levels. However, despite lower dietary phosphorus intake, hyperphosphatemia was still present in some malnourished patients, indicating the multifactorial nature of mineral disturbances in this population [54]. Similarly, in our study, the inverse association between MIS and phosphate levels may reflect both dietary inadequacies and the metabolic consequences of advanced malnutrition. Guida et al. demonstrated that dietary interventions, such as substituting protein with low-phosphorus formulations, could significantly reduce serum phosphate and calcium-phosphate product levels without compromising nutritional status [55]. In contrast, our study did not involve dietary interventions, but the observed increase in calcium-phosphate product in higher MIS quartiles suggests a need for more stringent dietary phosphate management in this subgroup. The findings emphasize that even modest dietary phosphorus control can mitigate biochemical disturbances, which could be an avenue for improving outcomes in patients with high MIS scores. Lynch et al. reported that more liberal dietary phosphate prescriptions were associated with better survival rates, especially in non-hyperphosphatemic patients, challenging the traditional emphasis on aggressive phosphate restriction [56]. In our study, lower phosphate levels in higher MIS quartiles might partially reflect reduced dietary intake and malnutrition rather than successful phosphate management. These results underscore the need to balance phosphorus restriction with adequate protein intake to avoid worsening malnutrition and its associated risks, particularly in high MIS quartile patients.

### Association between MIS and mental health conditions, and sleep quality

Our study investigated the association between MIS and mental health conditions, specifically depression, anxiety, and stress, using the Depression Anxiety Stress Scales (DASS-21). We found significant differences in depression scores across MIS quartiles, with participants in higher MIS quartiles reporting more severe depressive symptoms. Similarly, anxiety scores tended to be higher in patients with elevated MIS, although the association was not consistently significant. However, the trend for stress did not reach statistical significance, indicating that the relationship between MIS and stress may be weaker or inconsistent. Comparable to our findings, Ibrahim et al. noted a significant prevalence of depressive symptoms among hemodialysis patients, which was associated with reduced Quality of Life (QoL), overall symptom burden, and the malnutrition-inflammation complex [29]. Also, Koo et al. found that 38% of hemodialysis patients experienced significant depressive symptoms, with malnutrition being a key predictor [37]. Contrary to our findings, another study conducted on hemodialysis patients with cancer revealed that the correlation between MIS and depression, reduced quality of life, sleep disturbances, and Restless Leg Syndrome (RLS) was either absent or weak [30]. It is crucial to recognize that our findings indicate an association rather than causation between MIS and mental health conditions. The interplay between malnutrition, inflammation, and mental health is likely bidirectional, necessitating further longitudinal studies to establish causal pathways.

Chronic inflammation, common in hemodialysis patients with high MIS scores, can lead to alterations in neurotransmitter metabolism. Pro-inflammatory cytokines such as interleukin-6 (IL-6) and tumor necrosis factor-alpha (TNF-α) can influence the synthesis, release, and reuptake of neurotransmitters like serotonin, dopamine, and norepinephrine, which are critical for mood regulation [57, 58]. Malnutrition, as indicated by a high MIS score, can result in deficiencies of essential nutrients that are crucial for brain function, such as omega-3 fatty acids, B vitamins, and amino acids. These deficiencies can impair cognitive function and contribute to symptoms of depression and anxiety [59]. Inflammation and malnutrition can lead to dysregulation of the hypothalamic-pituitary-adrenal (HPA) axis, which plays a critical role in stress response. Chronic activation of the HPA axis can result in elevated cortisol levels, contributing to anxiety and depression [60]. Also, chronic illness and the stress of long-term hemodialysis treatment can lead to social isolation, reduced quality of life, and increased psychological stress, contributing to depression and anxiety [61, 62]. On the other hand, our findings regarding mental health conditions such as depression and anxiety emphasize the bidirectional relationship between malnutrition and mood disorders in hemodialysis patients [63, 64]. However, the non-significant trend observed for stress suggests that this relationship may not extend uniformly to all mental health conditions. Given the cross-sectional nature of our study, we cannot determine whether malnutrition contributes to depression and anxiety or whether these conditions lead to poor nutritional status. Further prospective studies are necessary to clarify these relationships.

Depression and low mood can lead to poor dietary intake, reduced motivation for self-care, and alterations in appetite-regulating hormones, all of which may contribute to the onset and progression of malnutrition [64, 65]. In turn, malnutrition may exacerbate depression through pathways such as chronic inflammation, neurotransmitter dysregulation, and fatigue, creating a vicious cycle. These observations highlight the importance of integrated nutritional and psychological interventions to break this cycle and improve patient outcomes [33].

Discrepancies in findings across studies may stem from differences in patient populations, mental health assessment tools, and cultural or regional contexts. For instance, cancer patients on hemodialysis may experience unique psychological stressors and disease burdens that could dilute the association between MIS and mental health. Additionally, the use of different scoring systems, such as the DASS-21 versus structured clinical interviews, might contribute to variations in sensitivity and specificity, leading to inconsistent results. Understanding these factors is essential for interpreting the association between MIS and mental health conditions.

These findings collectively highlight the complex interplay between malnutrition, inflammation, and mental health in hemodialysis patients, underscoring the importance of comprehensive mental health assessments and interventions in this population. However, due to the limitations of a cross-sectional design, caution must be exercised when interpreting the results, and further longitudinal research is warranted to confirm these associations and explore potential causal mechanisms. Moreover, the association between the MIS and mental health conditions may vary depending on the underlying health condition of hemodialysis patients, highlighting the need for further research to understand these complex relationships. Additionally, it is crucial to consider the underlying disease that necessitates hemodialysis and its impact on quality of life. This consideration facilitates the exploration of various approaches for managing treatment tailored to individual patients.

Analysis of sleep quality using the Pittsburgh Sleep Quality Index (PSQI) did not reveal significant differences across MIS quartiles. While some studies suggest a potential link between malnutrition, inflammation, and sleep disturbances in hemodialysis patients [28, 32, 66], our findings indicate that this association may be weak or inconsistent. The underlying mechanisms include chronic inflammation and its impact on neurotransmitter function, where pro-inflammatory cytokines like IL-6 and TNF-α disrupt normal sleep patterns by altering sleep architecture and increasing awakenings [67]. Malnutrition leads to deficiencies in essential nutrients such as magnesium, calcium, and vitamins B6 and B12, which are crucial for the production and regulation of melatonin and other sleep-related neurotransmitters [68]. Dysregulation of the hypothalamic-pituitary-adrenal (HPA) axis, resulting from chronic inflammation and malnutrition, elevates cortisol levels, thereby increasing arousal and hindering sleep [69]. Furthermore, the psychological stress associated with chronic illness and long-term hemodialysis exacerbates anxiety and depression, contributing to poor sleep quality [37]. However, the lack of a significant relationship in our study suggests that other factors, such as comorbidities, medication use (e.g., sleeping aids), or individual differences in sleep perception, may play a more substantial role in determining sleep quality among hemodialysis patients. Future research should explore these potential confounders through detailed subgroup analyses to gain a clearer understanding of the interplay between malnutrition, inflammation, and sleep quality. These findings highlight the importance of a comprehensive approach to patient management that considers both nutritional and psychological factors to optimize sleep and overall well-being in this population.

### Implications for clinical practice

The findings of this study underscore the need for personalized interventions to address malnutrition, inflammation, sleep disturbances, and mental health conditions in hemodialysis patients. Personalized care can be implemented using a risk stratification approach based on MIS scores and other relevant clinical parameters. Patients could be categorized into low, moderate, and high-risk groups, with targeted interventions tailored to each group’s specific needs.

Low-Risk Patients (MIS Score: 0–7): Focus on preventive measures to maintain nutritional and psychological well-being. This includes individualized dietary counseling, early mental health screenings, physical activity recommendations, and regular assessment of inflammatory markers to detect early signs of deterioration. Patients in this group should receive periodic education on protein-energy intake, fluid balance, and dietary phosphorus control to prevent progression toward malnutrition.

Moderate-Risk Patients (MIS Score: 8–18): Require multidisciplinary intervention involving dietitians, mental health professionals, nephrologists, and social workers to manage emerging nutritional and inflammatory issues. Dietary modifications should focus on optimizing protein intake while managing phosphorus levels through phosphate binders and low-phosphorus protein sources. Structured physical activity programs should be introduced to preserve muscle mass. Psychological support, including cognitive behavioral therapy (CBT), stress management, and, when necessary, low-dose antidepressants or anxiolytics, should be integrated to address early signs of depression and anxiety.

High-Risk Patients (MIS Score: 19–30): Require intensive, closely monitored interventions due to severe malnutrition, inflammation, and psychological distress. Nutritional support may involve enteral or parenteral nutrition to prevent further deterioration. Advanced psychological therapies, including psychotherapy and medication adjustments, should be considered for patients with severe depression or anxiety. Regular inflammation marker monitoring (e.g., C-reactive protein, IL-6) should guide anti-inflammatory interventions, including the use of omega-3 supplementation and anti-inflammatory dietary modifications. Close collaboration between nephrologists, dietitians, and mental health professionals is crucial, with biweekly or monthly multidisciplinary team meetings to adjust care plans dynamically.

To systematically implement personalized interventions, we propose an evaluation framework and decision-support tool that ensures standardized, individualized care:

Initial Assessment: Comprehensive evaluation using MIS, validated mental health tools (DASS-21, Beck Depression Inventory), and sleep quality assessments (PSQI).

Risk Stratification: Categorization into risk groups based on MIS scores, dialysis vintage, BMI, residual kidney function, and comorbidities.

Tailored Intervention Planning: Development of individualized care plans incorporating nutritional therapy, psychological support, physical rehabilitation, and pharmacologic interventions.

Monitoring and Reassessment: Regular follow-ups every 3 months (or more frequently for high-risk patients) to adjust dietary, medical, and psychological interventions based on patient progress.

This structured framework ensures evidence-based, adaptable interventions tailored to each patient’s clinical condition, improving overall care delivery and health outcomes in hemodialysis patients. The integration of this decision-support tool into routine practice can help clinicians identify high-risk patients earlier, prevent complications, and enhance patient-centered care.

### Strengths and limitations

The strengths of this study include its multicenter design, which allowed for the inclusion of a diverse patient population from different clinical settings. Additionally, the comprehensive assessment protocol employed in this study enabled a thorough evaluation of various health parameters, providing valuable insights into the complex interrelationships among malnutrition, inflammation, sleep quality, and mental health conditions in hemodialysis patients. Although the MIS score has established grading criteria, the use of quartiles in this study allowed for a more detailed exploration of the relationships between nutritional and inflammatory status and other health parameters. By dividing the MIS scores into quartiles, we captured variations that may be overlooked when using broader grading categories. This approach facilitated the identification of incremental trends and associations across the full range of MIS scores, enhancing the depth of analysis and interpretation. However, we acknowledge that using predefined categories aligns more closely with clinical practice and may be more intuitive for some readers. Future studies could compare the two methods to evaluate their relative utility in clinical and research settings.

Several limitations should be acknowledged. Firstly, the cross-sectional nature of the study precludes causal inference, and longitudinal studies are needed to establish temporal relationships between malnutrition, inflammation, sleep quality, and mental health conditions in hemodialysis patients. Secondly, the reliance on self-reported measures for assessing sleep quality and mental health conditions may introduce reporting biases and limit the generalizability of the findings. Future research should incorporate objective measures of sleep quality and mental health outcomes to enhance the validity of the results.

Another potential limitation is the inclusion of patients who recently used sleeping pills, which may have influenced the assessment of sleep quality. These medications could mask the true prevalence and severity of sleep disturbances, thereby introducing bias. Future studies should consider excluding patients using sleeping pills or analyzing this subgroup separately to better understand their impact on sleep quality assessments. Additionally, collecting detailed information on the type, dosage, and duration of sleeping pill usage would provide a deeper understanding of their influence on study outcomes.

Furthermore, dialysis-related factors, including vascular access type, may impact inflammation, nutrition, and sleep but were not fully assessed. Medications like antidepressants, sedatives, and phosphate binders could also affect sleep and mood, introducing potential confounders. Future studies should consider these factors for more robust findings.

Incomplete primary nephrological diagnosis data limited classification beyond diabetes and hypertension, and the lack of complete data on major comorbid conditions (MCC) was another limitation of this study. Future research with comprehensive data collection could address these gaps and provide a clearer understanding of the complex interplay between malnutrition, inflammation, sleep quality, and mental health in hemodialysis patients.

## Conclusion

In conclusion, this multicenter cross-sectional study provides valuable insights into the associations among malnutrition, inflammation, sleep quality, and mental health conditions in hemodialysis patients. While these findings highlight important relationships, it is essential to note that no causal relationship can be inferred due to the study’s cross-sectional design. These results underscore the need for comprehensive assessments and multidisciplinary approaches in patient care, as well as personalized interventions tailored to individual patient profiles. Addressing the diverse needs of hemodialysis patients may contribute to better patient outcomes and quality of life in this vulnerable population. Further research, particularly longitudinal studies, is warranted to clarify the underlying mechanisms of these associations and to inform the development of targeted interventions that support the overall health and well-being of individuals undergoing hemodialysis.

## Data Availability

The datasets used and/or analyzed during the current study available from the corresponding author on reasonable request.
